# Alkaline phosphatase of late pregnancy promotes the prediction of adverse birth outcomes

**DOI:** 10.7189/jogh.15.04028

**Published:** 2025-01-24

**Authors:** Bin Zhang, Zhaolong Zhan, Sijie Xi, Yinglu Zhang, Xiaosong Yuan

**Affiliations:** Department of Medical Genetics, Changzhou Maternal and Child Health Care Hospital, Changzhou Medical Centre, Nanjing Medical University, Changzhou, China

## Abstract

**Background:**

Adverse birth outcomes (ABO), such as preterm birth (PTB), small and large for gestational age (SGA/LGA), can compromise both the short- and long-term health of mothers and their foetuses. The purpose of this observational study was to investigate the association between maternal serum alkaline phosphatase (ALP) levels in late pregnancy and the risk of ABO, and to evaluate its predictive value of maternal ALP levels for ABO in women with singleton pregnancies.

**Methods:**

A total of 11 853 consecutive pregnant women underwent hepatic and renal function tests, lipid profile assessments, ALP and high-sensitivity C-reactive protein levels measurements upon admission for labour. Their clinical perinatal parameters and outcomes were also analysed.

**Results:**

The prevalence of PTB, SGA, and LGA in this study was 7.2% (n = 849), 8.9% (n = 1053), and 15.6% (n = 1844), respectively. With increasing quartiles of maternal serum ALP levels, the foetal gestational age increased by 0.58 weeks (95% confidence interval (CI) = 0.50–0.66), 0.78 weeks (95% CI = 0.70–0.86), and 0.98 weeks (95% CI = 0.90–1.06), respectively, and the birth weight increased by 62.91 g (95% CI = 43.96–81.86), 91.54 g (95% CI = 72.41–110.67), and 117.92 g (95% CI = 98.18–137.67), respectively. Compared to women in the bottom quartile of ALP, those in the top quartile had a lower risk of PTB (adjusted odds ratio (OR) = 0.14; 95% CI = 0.11–0.18), a lower risk of SGA (adjusted OR = 0.65; 95% CI = 0.53–0.80), and a higher risk of LGA (adjusted OR = 1.92; 95% CI = 1.62–2.28). Sensitivity analyses conducted among individuals without advanced maternal age, obesity, multiparity, pregnancy complications, and PTB (for SGA/LGA) validated the consistency of these results. More importantly, adding ALP to the established model significantly increased the area under the curve (AUC) for predicting adverse birth outcomes: for PTB, the AUC increased from 0.761 to 0.809 (*P* < 0.001); for SGA, it increased from 0.754 to 0.759 (*P* = 0.014); and for LGA, it increased from 0.750 to 0.755 (*P* < 0.001).

**Conclusions:**

Maternal serum ALP levels in late pregnancy are significantly associated with the risk of ABO. When combined with clinical characteristics and routine laboratory results, ALP has incremental predictive value for ABO, particularly for PTB.

In China, a three-tiered health service system is provided for women and children, consisting of community, district, and urban health care facilities. The main focus of this service system is on adverse birth outcomes (ABO), including preterm birth (PTB) and infants being small for gestational age (SGA) or large for gestational age (LGA) [1]. In China, the PTB rate ranges from 5.1 to 16.7%; in the USA and Europe, the reported rates are approximately 12–13% and 5–9%, respectively [2,3]. Preterm birth-linked complications are the primary causes of perinatal morbidity and mortality, and survivors are at greater risk of short- and long-term health issues [4]. Small for gestational age and LGA are also common threats to maternal and foetal health, with prevalences of 8.9 and 15.5%, respectively, in China [5]. Small for gestational age is an effective prognostic indicator of postnatal and neonatal morbidity, and children born SGA have an increased risk of growth retardation and neurologic dysfunction in childhood, as well as cardio-metabolic diseases in adulthood. Large for gestational age offspring also face greater risks of birth injuries, caesarean delivery, future obesity, diabetes, and cardiovascular diseases [6,7]. In an attempt to prevent complications related to these outcomes and improve maternal and foetal health, it is essential to explore the most accessible and effective indicators of ABO in late pregnancy, so that interventions can be targeted at women at higher risk.

Alkaline phosphatase (ALP) is a ubiquitous glycoprotein primarily derived from several tissues, including the liver, bone, small intestine, kidneys, and placenta. During pregnancy, ALP levels physiologically increase as pregnancy progresses, reaching a peak near the end of pregnancy, primarily due to contributions from placental and bone isoenzymes [8]. Abnormally higher ALP levels in pregnancy have been detected in various conditions, such as hypertensive disorders of pregnancy, gestational diabetes mellitus (GDM), intrahepatic cholestasis of pregnancy (ICP), PTB, intrauterine growth restriction, and low birth weight [9–17]. However, the association between high ALP levels and adverse pregnancy outcomes remains controversial, as several reports have shown that elevated ALP levels are not associated with these adverse outcomes [18–22]. For example, two retrospective case reports observed an association between high levels of maternal serum ALP and low birth weight [10,14]. Additionally, two other case reports demonstrated that pregnant women with extremely high serum ALP levels gave birth to foetuses with normal birth weight [21,22]. More notably, a prospective study involving 214 mothers and their offspring found an association between increased maternal serum ALP levels and LGA infants [23]. However, this study may have some limitations. For instance, the proportion of excluded GDM cases to the initial sample size was unreasonably high (233/544). Additionally, the remaining study samples failed to exclude the influence of other pregnancy complications on LGA, as well as the impact of other ABO, particularly SGA, on ALP levels.

Therefore, large observational cohort studies focusing on the impact of maternal ALP levels on foetal outcomes are urgently needed. This hospital-based study aimed to investigate the association between maternal serum ALP levels and ABO, as well as to assess the predictive value of ALP levels for ABO in women with singleton pregnancies within a homogeneous population that did not have pre-existing hepatorenal or skeletal diseases. A hospital-based cohort study is appropriate for addressing the research question because it allows for:

1) detailed and standardised data collection, ensuring accurate measurement of serum ALP levels and other biomarkers

2) a diverse sample population, providing a representative sample of pregnant women and enhancing generalisability

3) consistent data collection methods, which reduce bias and enhance the reliability and validity of the study results.

## METHODS

### Study design and data collection

An observational cohort study was conducted to include all consecutive women who gave birth between April 2016 and March 2017 at Changzhou Maternal and Child Health Care Hospital, a 3-A-class specialised medical facility in Southeast China. Information on maternal general characteristics (age, height, weight, blood pressure, unhealthy habits, medical history, reproductive history, and pregnancy complications) and offspring characteristics (gestational age, sex, birth length, and birth weight) was obtained from clinical records. Maternal laboratory results, including hepatic and renal function tests, lipid profiles, ALP levels, and high-sensitivity C-reactive protein (hsCRP) levels, were retrieved from the laboratory information systems of the hospital. The inclusion criteria for this study were as follows:

1) maternal age >18 years

2) singleton live births without birth defects.

The exclusion criteria were as follows:

1) smoking, using illicit drugs, or consuming alcohol during the current pregnancy

2) pre-existing pre-pregnancy diseases (such as type 1 or 2 diabetes mellitus, chronic hypertension, chronic cardiovascular, hepatorenal, or osteal diseases, immune rheumatic diseases, thyroid diseases, or syphilis)

3) lack of routine biochemical laboratory results (ALP, hsCRP, and hepatorenal function) upon hospital admission.

Among the 13 275 consecutive women observed, 1422 were excluded from this cohort study due to pre-pregnancy diseases (n = 488), multiple gestations (n = 335), non-live births (n = 96), and lack of biochemical laboratory results (n = 513). Eleven thousand eight hundred fifty-three eligible pregnant women were included in the final analysis. None of the eligible individuals consumed alcohol or illicit drugs or smoked during their current pregnancies. All participants in the present study were admitted to the hospital after experiencing symptoms of labour onset. Blood samples were collected before active labour. Laboratory tests on each sample were routinely performed using corresponding analytical methods and reagents on the same automated platforms: for ALP, hepatorenal function, and lipid profiles, the AU5800 from Beckman Coulter, Japan was used; for hsCRP, the BN II System from Siemens Diagnostics, Germany was utilised. The inter-assay and intra-assay coefficient of variation values for the laboratory analytes (ALP, hsCRP, hepatic and renal function tests, and blood lipids) were less than 5% and less than 10%, respectively.

This observational study was approved by the Ethics Committee of Changzhou Maternal and Child Health Care Hospital (Approval No. ZD201803) prior to data collection. The requirement for written informed consent was waived, as the data were retrospectively retrieved from the electronic medical system and analysed anonymously.

### Definitions of adverse birth outcomes

Foetal gestational age, sex, birth weight, and birth length were recorded by professional obstetricians and midwives. In this observational study, PTB, SGA, and LGA were considered the primary outcomes. Based on gestational age, the offspring were divided into two groups:

1) PTB, defined as deliveries at less than 37 weeks

2) full-term birth (FTB), defined as deliveries at 37 weeks or later [24].

According to birth weight, the offspring were classified into three categories:

1) SGA, defined using a Chinese reference curve as having a birth weight below the 10th percentile for their specific gestational week

2) appropriate for gestational age (AGA), defined as having a birth weight between the 10th and 90th percentiles

3) LGA, defined as having a birth weight above the 90th percentile [25].

### Statistical analysis

In this study, data were presented as mean ± standard deviation (SD) for continuous variables and as n (%) for categorical variables, stratified by maternal ALP quartile levels. Analysis of variance or Kruskal-Wallis tests were employed to compare differences in continuous variables across the quartiles, while χ^2^ or Fisher exact tests were used for categorical variables. Mann-Whitney tests were conducted to compare serum ALP levels between the PTB and FTB groups, and Kruskal-Wallis tests were used among the SGA, AGA, and LGA categories. Spearman’s correlation analyses investigated the correlations between ALP levels, maternal general characteristics, and other laboratory analytes. When treating foetal birth length, birth weight, and gestational week as continuous variables, general linear regression models were applied to determine their regression coefficients and 95% confidence intervals (CIs), assessing the impact of ALP on these variables. Logistic regression models, on the other hand, were used to calculate odds ratios (ORs) and 95% CIs, evaluating the magnitude of effects of ALP on categorical outcomes such as SGA, LGA, and PTB. Adjusted variables encompassed maternal age, body mass index (BMI), parity, blood pressure, pregnancy complications, assisted reproduction, foetal sex, and gestational age (specifically for birth length/birth weight and SGA/LGA analyses), along with other laboratory tests including blood lipids, hsCRP, and liver and kidney function indices. Sensitivity analyses among individuals without advanced maternal age, obesity, multiparity, pregnancy complications, and PTB (for SGA/LGA) were conducted to determine the reliability of the findings. Subgroup analyses explored interactions between categories of ALP levels and maternal age, BMI, parity, and foetal gestational week. Receiver operating characteristic (ROC) curve analyses were conducted to determine the optimal cutoff values of laboratory analytes and the best thresholds of different models for predicting ABO. Each parameter was evaluated using the maximum Youden index (sensitivity + specificity −1), and the area under the curve (AUC) was calculated to assess predictive power.

The statistical analyses were conducted using *R* (http://www.R-project.org, Vienna, Austria, 2024) and Empower Stats software (X&Y solutions, Boston, MA, USA, 2024), with *P* < 0.05 presented statistically significant.

## RESULTS

### Population characteristics

Of the 11 853 singleton live births, 7.2% (n = 849) were PTB, 8.9% (n = 1053) were SGA, and 15.6% (n = 1844) were LGA. The mean (SD) foetal birth length, birth weight, and gestational age were 49.8 (1.4) cm, 3339.1 (498.7) g, and 38.7 (1.7) weeks, respectively. The mean (SD) maternal age and BMI at the time of hospitalisation for delivery were 28.6 (4.4) years and 27.3 (3.4) kg/m^2^, respectively. Sixty percent of the women were nulliparous. The prevalences of GDM, ICP, preeclampsia (PE), and pregnancy induced hypertension (PIH) in the study population were 8.4% (n = 1000), 6.2% (n = 733), 3.6% (n = 427), and 2.1% (n = 251), respectively.

The general characteristics of mothers and their offspring are summarised in **Table 1** by ALP quartiles (Q): Q1 < 122 U/L; Q2 = 122–148 U/L; Q3 = 149–182 U/L; and Q4 > 182 U/L. Significant differences among the ALP quartile groups were observed for demographic characteristics and laboratory results, with the exception of the assisted reproduction rate, PE incidence, and triglyceride, high-density lipoprotein cholesterol (HDL-C), and hsCRP levels. There was a step-wise decrease in the incidence of PTB (15.3 vs. 6.2 vs. 4.0 vs. 3.4%, *P* < 0.001), and a step-wise increase in foetal birth weight (3179.9 ± 574.2 vs. 3351.1 ± 472.8 vs. 3398.8 ± 454.3 vs. 3422.9 ± 448.5 g, *P* < 0.001) and gestational age (38.0 ± 2.2 vs. 38.7 ± 1.5 vs. 38.9 ± 1.4 vs. 39.1 ± 1.3 weeks, *P* < 0.001) with increasing ALP quartile. Compared with women in the Q1 of ALP, those in the Q4 had a higher incidence of delivering LGA offspring (16.3 vs. 13.9%) and a lower incidence of delivering SGA offspring (8.3 vs. 10.3%, *P* = 0.009).

**Table 1:** General characteristics of 11 853 mothers and their offspring by serum ALP level quartiles*

Variables	ALP (U/L)				*P*-value
	**Q1 (<122, n = 2927)**	**Q2 (122–148, n = 2914)**	**Q3 (149–182, n = 2995)**	**Q4 (>182, n = 3017)**	
**Maternal characteristics**					
Age (years)	29.5 ± 4.5	28.9 ± 4.4	28.3 ± 4.4	27.8 ± 4.3	<0.001
*<20*	14 (0.5%)	21 (0.7%)	32 (1.1%)	46 (1.5%)	<0.001
*20–34*	2616 (89.4%)	2638 (90.5%)	2719 (90.8%)	2786 (92.3%)	<0.001
*≥35*	297 (10.1%)	255 (8.8%)	244 (8.1%)	185 (6.1%)	<0.001
BMI (kg/m^2^)†	27.8 ± 3.5	27.6 ± 3.4	27.2 ± 3.2	26.8 ± 3.2	<0.001
*<25*	619 (21.5%)	664 (23.0%)	765 (25.7%)	880 (29.4%)	<0.001
*25–29*	1548 (53.7%)	1572 (54.5%)	1669 (56.2%)	1638 (54.7%)	<0.001
*≥30*	714 (24.8%)	646 (22.4%)	537 (18.1%)	476 (15.9%)	<0.001
**Parity**					
No child	1486 (50.8%)	1703 (58.4%)	1878 (62.7%)	2043 (67.7%)	<0.001
≥1 child	1441 (49.2%)	1211 (41.6%)	1117 (37.3%)	974 (32.3%)	<0.001
**Systolic BP (mmHg)**	120.6 ± 12.9	120.7 ± 12.1	120.9 ± 11.8	121.8 ± 11.9	<0.001
**Diastolic BP (mmHg)**	74.2 ± 8.8	74.2 ± 8.3	74.6 ± 8.1	75.1 ± 8.3	<0.001
**Gestational age (week)**	38.0 ± 2.2	38.7 ± 1.5	38.9 ± 1.4	39.1 ± 1.3	<0.001
**Assisted reproduction**	74 (2.5%)	82 (2.8%)	66 (2.2%)	57 (1.9%)	0.102
**Delivery mode**					
Vaginal delivery	1447 (49.4%)	1590 (54.6%)	1811 (60.5%)	1931 (64.0%)	<0.001
Caesarean section	1480 (50.6%)	1324 (45.4%)	1184 (39.5%)	1086 (36.0%)	<0.001
**Pregnancy complications**‡					
GDM	290 (9.9%)	245 (8.4%)	241 (8.0%)	224 (7.4%)	0.005
ICP	131 (4.5%)	149 (5.1%)	165 (5.5%)	288 (9.5%)	<0.001
PE	124 (4.2%)	100 (3.4%)	94 (3.1%)	109 (3.6%)	0.139
PIH	64 (2.2%)	48 (1.6%)	58 (1.9%)	81 (2.7%)	0.040
PTB	448 (15.3%)	180 (6.2%)	119 (4.0%)	102 (3.4%)	<0.001
**Newborn characteristics**					
Sex					
*Female*	1572 (53.7%)	1458 (50.0%)	1352 (45.1%)	1212 (40.2%)	<0.001
*Male*	1355 (46.3%)	1456 (50.0%)	1643 (54.9%)	1805 (59.8%)	<0.001
Birth length (cm)	49.4 ± 2.1	49.9 ± 1.3	49.9 ± 1.1	50.0 ± 0.9	<0.001
Birth weight (g)	3179.9 ± 574.2	3351.1 ± 472.8	3398.8 ± 454.3	3422.9 ± 448.5	<0.001
Weight for gestational age					
*SGA*	302 (10.3%)	259 (8.9%)	242 (8.1%)	250 (8.3%)	0.009
*AGA*	2217 (75.7%)	2190 (75.2%)	2274 (75.9%)	2275 (75.4%)	0.009
*LGA*	408 (13.9%)	465 (16.0%)	479 (16.0%)	492 (16.3%)	0.009
**Laboratory tests**					
Total bilirubin (μmol/L)	7.7 ± 3.0	7.8 ± 2.9	8.0 ± 3.0	8.1 ± 3.1	<0.001
Direct bilirubin (μmol/L)	1.5 ± 0.9	1.5 ± 0.9	1.6 ± 0.9	1.7 ± 1.3	<0.001
ALT (U/L)	11.0 ± 13.0	10.7 ± 7.5	10.8 ± 7.6	13.9 ± 21.7	<0.001
AST (U/L)	19.4 ± 24.8	19.3 ± 7.2	19.5 ± 6.1	22.4 ± 17.3	<0.001
γ-GT (U/L)	12.2 ± 7.7	12.8 ± 9.9	12.9 ± 8.5	16.0 ± 19.3	<0.001
Total protein (g/L)	62.9 ± 4.3	63.4 ± 4.3	63.7 ± 4.4	64.0 ± 4.5	<0.001
Albumin (g/L)	36.3 ± 2.5	36.3 ± 2.5	36.5 ± 2.5	36.5 ± 2.7	0.009
Urea nitrogen (mmol/L)	3.5 ± 1.0	3.6 ± 0.9	3.5 ± 0.9	3.6 ± 0.9	0.011
Creatinine (umol/L)	58.7 ± 9.0	59.8 ± 8.1	60.4 ± 8.1	61.5 ± 10.2	<0.001
Total cholesterol (mmol/L)	6.2 ± 1.2	6.3 ± 1.2	6.4 ± 1.2	6.5 ± 1.2	<0.001
Triglyceride (mmol/L)	3.9 ± 1.8	4.0 ± 1.9	4.0 ± 1.8	3.9 ± 1.7	0.283
LDL-C (mmol/L)	3.2 ± 0.9	3.3 ± 0.9	3.4 ± 0.9	3.5 ± 0.9	<0.001
HDL-C (mmol/L)	1.7 ± 0.3	1.7 ± 0.3	1.7 ± 0.3	1.7 ± 0.4	0.063
hsCRP (mg/L)	4.2 ± 5.6	4.4 ± 6.2	4.3 ± 5.6	4.4 ± 6.5	0.335

AGA – appropriate for gestational age, ALP – alkaline phosphatase, ALT – alanine aminotransferase, AST – aspartate aminotransferase, BMI – body mass index, BP – blood pressure, GDM – gestational diabetes mellitus, HDL-C – high density lipoprotein cholesterol, hsCRP – high sensitive C-reactive protein, ICP – intrahepatic cholestasis of pregnancy, LDL-C – low density lipoprotein cholesterol, LGA – large for gestational age, PE – pre-eclampsia, PIH – pregnancy induced hypertension, PTB – preterm birth, Q – quartile, SGA – small for gestational age, γ-GT – r-glutamyl transpeptidase

*Data were expressed as mean ± SD and n (%).

†125 participants lacked of BMI due to height loss.

‡234 participants developed two types of pregnancy complications.

### Serum ALP and maternal characteristics

The median (range) maternal ALP level at the time of hospitalisation for delivery was 149 U/L (12–1832 U/L). The distribution of ALP levels according to birth outcomes is shown in Table S1 in the **Online Supplementary Document**. Compared with women with FTB, maternal serum ALP levels at the time of hospitalisation were remarkably lower in those with PTB (median 118 vs. 151 U/L, *P* < 0.001). Serum ALP levels in women who delivered SGA/LGA offspring were significantly different from those in women who delivered AGA offspring (median: 145/151 U/L vs. 149 U/L, all *P* < 0.001). In addition, ALP was positively correlated with maternal blood pressure, foetal gestational age, liver and kidney function (except for albumin), total cholesterol, and LDL-C, and negatively correlated with maternal age, BMI, parity, and HDL-C (Table S2 in the **Online Supplementary Document**).

### Serum ALP and foetal growth and adverse birth outcomes

**Table 2** presents the impact of maternal ALP levels on offspring growth. With increasing ALP quartile, foetal birth length, birth weight, and gestational age increased by 0.50, 0.56, and 0.63 cm, 171, 219, and 243 g and 0.66, 0.88, and 1.03 week, respectively. After adjustment for confounding variables, these associations persisted. Compared with the Q1 of ALP, being in the Q4 reduced the risk of PTB and SGA by 86 and 35%, and increased the risk of LGA by 92% after controlling for the selected variables: PTB (adjusted OR = 0.14; 95% CI = 0.11–0.18; *P* < 0.001), SGA (adjusted OR = 0.65; 95% CI = 0.53–0.80, *P* < 0.001), LGA (adjusted OR = 1.92; 95% CI = 1.62–2.28, *P* < 0.001 (**Table 3**). Consistent findings were also noticed in the sensitivity analyses among participants without advanced maternal age (Table S3 in the **Online Supplementary Document**), obesity (Table S4 in the **Online Supplementary Document**), multiparity (Table S5 in the **Online Supplementary Document**), pregnancy complications (Table S6 in the **Online Supplementary Document**), and PTB (for SGA/LGA, Table S7 in the **Online Supplementary Document**). Grouping of the variables such as maternal age, BMI, parity, and foetal gestational week (for SGA/LGA) did not affect the associations between ALP levels and the risk of ABO (Table S8 and Table S9 in the **Online Supplementary Document**, all *P* for interaction >0.05).

**Table 2:** Regression coefficients β (95% CI) for foetal growth associated with quartiles of serum ALP level

Models	Gestational age (weeks)		Birth length (cm)§		Birth weight (g)§	
	**β (95% CI)**	***P-*value**	**β (95% CI)**	***P-*value**	**β (95% CI)**	***P-*value**
Model 1*						
*Q1 (<122 U/L)*	0		0		0	
*Q2 (122–148 U/L)*	0.66 (0.58–0.75)	<0.001	0.50 (0.43–0.58)	<0.001	171.21 (146.10–196.32)	<0.001
*Q3 (149–182 U/L)*	0.88 (0.80–0.96)	<0.001	0.56 (0.49–0.63)	<0.001	218.86 (193.92–243.81)	<0.001
*Q4 (>182 U/L)*	1.03 (0.95–1.12)	<0.001	0.63 (0.56–0.70)	<0.001	242.99 (218.09–267.89)	<0.001
P for trend		<0.001		<0.001		<0.001
Model 2†						
*Q1 (<122 U/L)*	0		0		0	
*Q2 (122–148 U/L)*	0.61 (0.53–0.69)	<0.001	0.07 (0.02–0.12)	0.003	58.94 (39.74–78.15)	<0.001
*Q3 (149–182 U/L)*	0.83 (0.75–0.91)	<0.001	0.06 (0.01–0.11)	0.015	92.06 (72.72–111.40)	<0.001
*Q4 (>182 U/L)*	1.00 (0.92–1.08)	<0.001	0.12 (0.07–0.17)	<0.001	113.53 (93.83–133.24)	<0.001
P for trend		<0.001		<0.001		<0.001
Model 3‡						
*Q1 (<122–U/L)*	0		0		0	
*Q2 (122–148 U/L)*	0.58 (0.50–0.66)	<0.001	0.08 (0.04–0.13)	<0.001	62.91 (43.96–81.86)	<0.001
*Q3 (149–182 U/L)*	0.78 (0.70–0.86)	<0.001	0.06 (0.01–0.11)	0.012	91.54 (72.41–110.67)	<0.001
*Q4 (>182 U/L)*	0.98 (0.90–1.06)	<0.001	0.13 (0.08–0.18)	<0.001	117.92 (98.18–137.67)	<0.001
P for trend		<0.001		<0.001		<0.001

ALP – alkaline phosphatase, BMI – body mass index, BP – blood pressure, CI – confidence interval, hsCRP – high sensitive C-reactive protein, Q – quartile

*Unadjusted.

†Adjusted for maternal age, BMI, parity, foetal gestational age, systolic and diastolic BP, pregnancy complications, assisted reproduction and foetal sex.

‡Adjusted for Model 2 variables plus blood lipids, hsCRP, liver and kidney function.

§Additionally corrected for foetal gestational age.

**Table 3:** ORs and 95% CIs for adverse birth outcomes associated with quartiles of serum ALP levels

Models	PTB		SGA§		LGA§	
	**OR (95% CI)**	***P*-value**	**OR (95% CI)**	***P*-value**	**OR (95% CI)**	***P*-value**
Model 1*						
*Q1 (<122 U/L)*	1		1		1	
*Q2 (122–148 U/L)*	0.36 (0.30–0.44)	<0.001	0.87 (0.73–1.04)	0.116	1.18 (1.02–1.37)	0.026
*Q3 (149–182 U/L)*	0.23 (0.19–0.28)	<0.001	0.78 (0.65–0.93)	0.007	1.18 (1.02–1.37)	0.024
*Q4 (>182 U/L)*	0.19 (0.16–0.24)	<0.001	0.81 (0.68–0.96)	0.018	1.24 (1.07–1.43)	0.004
*P for trend*		<0.001		0.015		0.009
Model 2†						
*Q1 (<122 U/L)*	1		1		1	
*Q2 (122–148 U/L)*	0.35 (0.29–0.42)	<0.001	0.88 (0.73–1.07)	0.198	1.35 (1.15–1.57)	<0.001
*Q3 (149–182 U/L)*	0.20 (0.16–0.26)	<0.001	0.74 (0.61–0.90)	0.002	1.57 (1.34–1.84)	<0.001
*Q4 (>182 U/L)*	0.15 (0.12–0.19)	<0.001	0.70 (0.57–0.85)	<0.001	1.88 (1.60–2.21)	<0.001
*P for trend*		<0.001		<0.001		<0.001
Model 3‡						
*Q1 (<122 U/L)*	1		1		1	
*Q2 (122–148 U/L)*	0.35 (0.28–0.43)	<0.001	0.85 (0.70–1.03)	0.098	1.39 (1.18–1.63)	<0.001
*Q3 (149–182 U/L)*	0.21 (0.17–0.27)	<0.001	0.71 (0.58–0.86)	<0.001	1.59 (1.35–1.88)	<0.001
*Q4 (>182 U/L)*	0.14 (0.11–0.18)	<0.001	0.65 (0.53–0.80)	<0.001	1.92 (1.62–2.28)	<0.001
*P for trend*		<0.001		<0.001		<0.001

ALP – alkaline phosphatase, BMI – body mass index, BP – blood pressure, CI – confidence interval, hsCRP – high sensitive C-reactive protein, LGA – large for gestational age, OR – odds ratio, PTB – preterm birth, Q – quartile, SGA – small for gestational age

*Unadjusted.

†Adjusted for maternal age, BMI, parity, foetal gestational age, systolic and diastolic BP, pregnancy complications, assisted reproduction and foetal sex.

‡Adjusted for Model 2 variables plus blood lipids, hsCRP, liver and kidney function.

§Additionally corrected for foetal gestational age.

### Serum ALP and prediction of adverse birth outcomes

**Figure 1:**
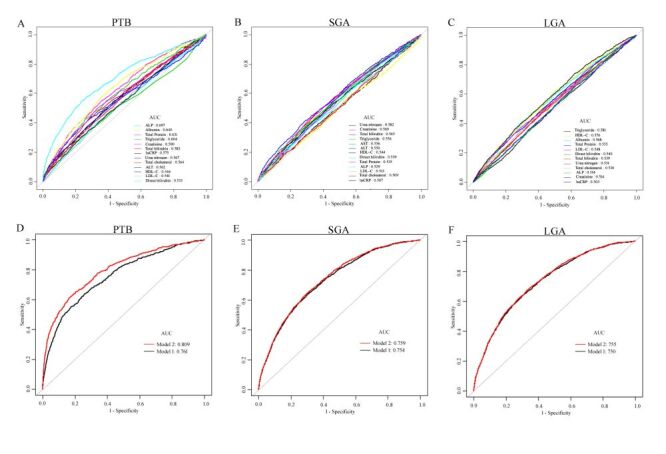
Receiver operating characteristic (ROC) curves to appraise the performance of different variables and models in predicting PTB and SGA/LGA. **Panel A.** PTB. **Panel B.** SGA. **Panel C.** LGA. **Panel D.** PTB. **Panel E.** SGA. **Panel F.** LGA. Model 1: maternal age, height, weight, gravidity, parity, blood BP, pregnancy complications, assisted reproduction, foetal sex, blood lipids, liver and kidney function and hs-CRP. Model 2: Model 1 plus ALP. ALP – alkaline phosphatase, AUC – area under the curve, BP – blood pressure, hsCRP – high sensitive C-reactive protein, LGA – large for gestational age, PTB – preterm birth, SGA – small for gestational age.

Receiver operating characteristic curves are drawn to investigate the predictive ability of ALP and other variables in detecting the risk of ABO (**Figure 1**, Panels A–F). The AUC of ALP for PTB was 0.697 (Table S10 in the **Online Supplementary Document**), which was remarkably larger than those of the other parameters. The best cut-off value of ALP for predicting PTB was 126.5 U/L, with a sensitivity of 57.8% and a specificity of 73.1%, and positive predictive value and negative predictive value of 14.2 and 95.7%, respectively. However, ALP did not show a better predictive value than the other parameters in identifying SGA or LGA risk (Table S11 and Table S12 in the **Online Supplementary Document**). To build logistic prediction models for ABO at the time of hospitalisation, all available clinical characteristics and laboratory results were integrated. Model 1 included maternal age, height, weight, parity, blood pressure, pregnancy complications, assisted reproduction, foetal sex, blood lipids, liver and kidney function, hsCRP level, and foetal gestational week (for SGA/LGA prediction). The AUC for PTB in Model 1 was 0.761 (95% CI = 0.743–0.780). Introducing ALP into the Model 1 remarkably increased the AUC to 0.809 (95% CI = 0.791–0.826, *P* < 0.001) (Table S10 in the **Online Supplementary Document**). Additionally, the incorporation of ALP slightly increased the AUC for detecting SGA delivery from 0.754 to 0.759 (*P* = 0.014,) (Table S11 in the **Online Supplementary Document**) and for LGA delivery from 0.750 to 0.755 (*P* < 0.001) (Table S12 in the **Online Supplementary Document**).

## DISCUSSION

In the present hospital-based observational study, we noted that maternal serum ALP levels measured at the time of admission for labour was positively associated with foetal gestational age, birth weight, and the LGA risk, and inversely associated with the risks of PTB and SGA. In addition, ALP level had a higher AUC for predicting PTB than other indices of inflammation, hepatic and renal function, and blood lipids. More importantly, combining ALP with maternal clinical characteristics and laboratory results significantly optimised the prediction of ABO, particularly PTB.

In general, ALP measured in the blood is a diagnostic tool for liver and bone diseases. During pregnancy, increased ALP activity from placental production is a normal variant that facilitates foetal growth and development [26]. It appears routinely in clinical laboratory tests. However, normal reference curves for pregnant women in different trimesters of pregnancy remain remains unclear. Several previous studies and a meta-analysis concerning reference intervals for pregnant women were based on smaller sample sizes, ranging from 52 to 565 women [27–31]. In two recent studies, Titaux et al. and Dai et al. established the reference curves for every gestational week (2–41 weeks) and sequential periods (6–7 weeks) of over 2000 pregnancies in France and China, respectively [8,32]. While ALP levels increase in late pregnancy, none of the previous studies with large samples have established reference intervals for the entire trimester. In the present cohort study, we described in detail the maternal distribution of serum ALP levels in accordance with pregnancy outcomes in 11 853 Chinese women. For this study population, the ALP reference intervals (median and the 5–95th percentile) for late pregnancy were 149 (89–248 U/L) for 9676 NPC women and 151 (92–252 U/L) for 11 004 FTB women. The present findings contribute information to the reference values for serum ALP in the third trimester of pregnancy. Additionally, we showed that serum ALP levels significantly increased with ICP and PIH (NPC vs. ICP/PIH median = 149 U/L vs. 166/156 U/L; all *P* < 0.05), which is in accordance with the previous studies [8,15–17].

High serum ALP levels are correlated with ABO, although this correlation is debated for both PTB and unfavourable birth weight. Several reports have demonstrated the risks of PTB, intrauterine growth restriction, and low birth weight neonates associated with elevated ALP levels, although fewer studies have conversely shown that decreased ALP levels were associated with PTB and intrauterine growth retardation [8–10,14,20,26]. In this study, maternal serum ALP levels measured upon admission for labour were inversely associated with the risks of PTB and SGA. We speculate that this discrepancy may be explained by the differences in study populations and/or stages of pregnancy, study designs, collection times for blood samples, and defining standards of high ALP levels [33]. For example, Goldenberg et al. demonstrated associations between maternal ALP levels and ABO in 580 pregnant women at 19 and 26 gestational weeks. They did not find a significant association with PTB when the measurements were performed at the 19th week. However, at 26 gestational weeks, they observed that high ALP levels were associated with an increased risk for PTB, and lower birth weight [34]. Additionally, the discrepancy could be due to a failure to control for multiple confounding factors regarding the associations of maternal ALP levels with ABO in some studies, whereas the present study was adjusted for detailed pregnancy complications. In the present study, elevated serum ALP levels at hospital admission for labour were independently associated with higher foetal gestational week and birth weight, and therefore, increased LGA and decreased PTB and SGA incidence. Our results confirm and extend the finding of a previous report that showed a significantly positive association between ALP levels and LGA risk during normal pregnancy [23].

Developing a tool that can identify pregnant women at risk of ABO early would be of great utility, as women could be provided with a personalised management and monitoring scheme tailored to their risk level. In this study, although ALP was significantly superior to other routine biochemical indices in predicting PTB risk, better performance was achieved when both biochemical parameters and clinical characteristics were used jointly. However, when using ALP as a sole screening marker for PTB, it should be noted that the low sensitivity of this marker may lead to the potential under-detection of women at risk of PTB. Additionally, the ability of ALP to predict SGA/LGA risk was significantly lower than that of other parameters. The prediction of PTB and SGA/LGA based on clinical characteristics and biochemical indicators routinely collected in clinical practice might be further improved. Further research is urgently required to validate our findings in diverse populations, to integrate ALP with advanced imaging techniques such as ultrasound and magnetic resonance imaging (MRI), to collect detailed data on lifestyle and environmental factors, to investigate psychosocial and genetic factors, to use advanced statistical methods such as propensity score matching and machine learning, and to conduct clinical trials to evaluate interventions aimed at modulating ALP levels or addressing underlying causes.

Our findings demonstrated a positive association between maternal serum ALP level, foetal gestational age, and birth weight. However, the exact mechanism underlying the relationship between ALP levels in late pregnancy and ABO risk remains unclear. At the end of mid-pregnancy, maternal ALP levels significantly increase, largely due to placental production, which has been implicated in placental differentiation, nutrient transportation, and regulation of maternal/foetal metabolism [35]. Maternal high ALP levels from the placenta are a normal variant of late pregnancy to maintain foetal growth and development [26]. We speculate that placental dysfunction linked to abnormal ALP level among pregnant women with unfavourable foetal growth may be involved in an increased risk of ABO [36,37].

This observational study is one of the few focusing on maternal ALP levels at admission for labour associated with birth weight and the incidence of SGA/LGA offspring. Moreover, we assessed other indices of hepatic and renal function, blood lipids, and inflammation, allowing comparisons of their predictive performance as predictors of ABO. The advantages of this study are as follows:

1) adequate samples of women with high and low serum ALP levels and sufficient statistical power to develop various predictive models for ABO, making our findings suitable for clinical settings

2) maternal laboratory results and birth outcomes were prospectively recorded in the database

3) most of the potential confounding variables were adjusted in the regression analysis.

However, this study has several limitations. First, since this is a post-hoc analysis, we cannot completely eliminate residual confounding, even though we adjusted for all available potential confounding factors in the final analyses. Residual confounding from unmeasured variables, such as lifestyle factors, socioeconomic status, environmental exposures, psychosocial stress, and genetic factors, can significantly influence the associations between ALP and ABO. Second, in the present study, ALP isozymes were not measured because total ALP is a routine parameter and is widely used in most hospitals in China. However, different ALP isozymes may have distinct biological roles and associations with pregnancy outcomes [38]. Placental ALP isoenzymes, which are specifically produced during pregnancy, may have a stronger association with pregnancy-related complications compared to other isozymes [37]. In the overwhelming majority of reported studies, if other isoenzymes can be determined, placental isoenzymes are often found to be the main fraction. Identifying placental ALP isoenzymes and pathology results would lay a solid foundation for this study. Excluding data on placental ALP isoenzymes could potentially underestimate the true relationship between ALP and ABO. Third, our study did not consider other factors affecting ALP levels, such as medication use and dietary factors, and other causes that may lead to hepatorenal and bone diseases during pregnancy, which may potentially influence the association between ALP and ABO. Finally, some statistical methods were not applied in our statistical analysis, which may affect the accuracy of the results. For example, in performing the AUC analysis and calibration plot evaluation, the data was not balanced. Despite the large sample size of this study, even if there is data imbalance, its impact on the predictive model's performance is likely to be minimal. In such cases, the necessity of balancing the data decreases. Except for 125 participants who were missing height data and thus could not have their BMI calculated, the data for the remaining participants were complete. In our study, we decided not to perform BMI value imputation because of the sufficiently large sample size. Given the varying incidence rates of ABO across different populations, caution should be exercised when generalising the findings of this study to other populations. Additionally, laboratory measurements for the participants were taken upon admission for labour (28–41 gestational weeks) rather than at a consistent gestational week. Although we controlled for gestational week in the statistical analyses and conducted sensitivity analyses among FTB participants, the impact of varying gestational weeks on the association of ALP levels with ABO cannot be completely excluded. A well-designed, population-based, multi-centre prospective study could offer a more compelling perspective on the association between maternal serum ALP levels and ABO incidence, as well as the predictive value of ALP in ABO risk assessment.

## CONCLUSIONS

This large observational study confirmed that the maternal serum ALP level measured upon admission for labour is a significant predictor of ABO. Furthermore, incorporating ALP levels into convenient and cost-effective models, which include maternal clinical characteristics and routine laboratory results, has an incremental predictive value for ABO, especially for PTB. By integrating ALP with other markers and adopting a comprehensive risk assessment strategy, including more efficient triage systems and targeted interventions for high-risk populations, policymakers and clinicians can better address the challenges of PTB and improve maternal and neonatal health.

## Additional material


Online Supplementary Document
