# Supplementary material for: Alkaline phosphatase of late pregnancy promotes the prediction of adverse birth outcomes
Source: J Glob Health. 2025 Jan 24;15:04028. doi: 10.7189/jogh.15.04028 (PMC11758466; doi:10.7189/jogh.15.04028)
Supplement: Online Supplementary Document [file jogh-15-04028-s001.pdf]

Table S1. The distribution of serum ALP levels at hospitalisation for labour according to different birth outcomes

**Variables**	**N**	**P 5**	**P 10**	**P 25**	**Median**	**P 75**	**P 90**	**P 95**	***P*-value**
All women*	11 853	89	101	122	149	183	222	251	
NPC	9676	89	101	122	149	182	220	248	
GDM	1000	82	94	118	145	178	215	246	0.298†
ICP	733	98	108	133	166	209	258	298	< 0.001†
PE	427	80	95	116	144	183	222	261	0.493†
PIH	251	94	104	121	156	197	239	273	0.026†
FTB	11 004	92	104	124	151	185	224	252	
PTB	849	65	75	95	118	150	191	219	< 0.001‡
AGA	8956	89	101	122	149	183	222	251	
SGA	1053	84	95	116	145	181	224	263	< 0.001§
LGA	1844	90	105	125	151	185	222	246	< 0.001§

AGA – appropriate for gestational age, ALP – alkaline phosphatase, FTB – full term birth, GDM – gestational diabetes mellitus, ICP – intrahepatic cholestasis of pregnancy, LGA – large for gestational age, NPC – no pregnant complications, P – percentile, PE – pre-eclampsia, PIH – pregnancy induced hypertension, PTB – pre-term birth, SGA – small for gestational age.

*234 participants developed two types of pregnancy complications.

†Compared to NPC group.

‡Compared to FTB group.

§Compared to AGA group.

Table S2. Correlation between ALP level and general characteristics at hospitalisation for labour

**Variable**	***r***	***P*-value**
Age	−0.153	< 0.001
BMI	−0.111	< 0.001
Systolic BP	0.047	< 0.001
Diastolic BP	0.051	< 0.001
Parity	−0.132	< 0.001
Foetal gestational age	0.201	< 0.001
Total bilirubin (μmol/L)	0.058	< 0.001
Direct bilirubin (μmol/L)	0.055	< 0.001
ALT (U/L)	0.030	< 0.001
AST (U/L)	0.144	< 0.001
γ-GT	0.097	< 0.001
Total protein (g/L)	0.096	< 0.001
Albumin (g/L)	0.026	0.089
Urea nitrogen (mmol/L)	0.026	< 0.001
Creatinine (μmol/L)	0.123	< 0.001
Total cholesterol (mmol/L)	0.102	< 0.001
Triglyceride (mmol/L)	0.004	0.694
LDL-C (mmol/L)	0.127	< 0.001
HDL-C (mmol/L)	−0.020	0.033
hsCRP (mg/L)	−0.003	0.731

ALP – alkaline phosphatase, ALT – alanine aminotransferase, AST – aspartate aminotransferase, BMI – body mass index, BP – blood pressure, HDL-C – high density lipoprotein cholesterol, hsCRP – high sensitive C-reactive protein, LDL-C – low density lipoprotein cholesterol, γ-GT – r-glutamyl transpeptidase.

Table S3. ORs and 95 % CIs for adverse birth outcomes associated with quartiles of serum ALP levels among individuals with non-advantage (n=10,477)

**Variables**	PTB		SGA§		LGA§	
	OR (95 % CI)	***P*-value**	OR (95 % CI)	***P*-value**	OR (95%CI)	***P*-value**
Model 1*						
Q1 (<122 U/L)	1		1		1	
Q2 (122–148 U/L)	0.36 (0.30, 0.44)	<0.001	0.88 (0.73, 1.07)	0.198	1.22 (1.03, 1.43)	0.018
Q3 (149–182 U/L)	0.23 (0.19, 0.29)	<0.001	0.80 (0.66, 0.97)	0.023	1.23 (1.05, 1.45)	0.011
Q4 (>182 U/L)	0.19 (0.15, 0.24)	<0.001	0.82 (0.68, 0.99)	0.040	1.24 (1.05, 1.45)	0.009
P for trend		<0.001		0.036		0.024
Model 2†						
Q1 (<122 U/L)	1		1		1	
Q2 (122–148 U/L)	0.34 (0.28, 0.42)	<0.001	0.86 (0.71, 1.04)	0.124	1.44 (1.21, 1.71)	<0.001
Q3 (149–182 U/L)	0.21 (0.17, 0.27)	<0.001	0.73 (0.60, 0.90)	0.003	1.65 (1.39, 1.97)	<0.001
Q4 (>182 U/L)	0.15 (0.12, 0.20)	<0.001	0.71 (0.58, 0.87)	0.001	1.87 (1.56, 2.23)	<0.001
P for trend		<0.001		0.001		<0.001
Model 3‡						
Q1 (<122 U/L)	1		1		1	
Q2 (122–148 U/L)	0.34 (0.27, 0.42)	<0.001	0.82 (0.67, 1.01)	0.060	1.48 (1.24, 1.78)	<0.001
Q3 (149–182 U/L)	0.21 (0.17, 0.27)	<0.001	0.69 (0.56, 0.85)	0.001	1.67 (1.39, 2.01)	<0.001
Q4 (>182 U/L)	0.13 (0.10, 0.17)	<0.001	0.64 (0.51, 0.79)	<0.001	1.94 (1.61, 2.34)	<0.001
P for trend		<0.001		<0.001		<0.001

ALP – alkaline phosphatase, PTB – preterm birth, SGA – small for gestational age, LGA – large for gestational age, OR – odds ratio, CI – confidence interval, Q – quartile, BMI – body mass index, BP – blood pressure, hsCRP – high sensitive C-reactive protein

*Unadjusted.

†Adjusted for maternal age, BMI, parity, fetal gestational age, systolic and diastolic BP, pregnancy complications, assisted reproduction and fetal sex.

‡Adjusted for Model 2 variables plus blood lipids, hsCRP, liver and kidney function.

§Additionally corrected for fetal gestational age.

Table S4. ORs and 95 % CIs for adverse birth outcomes associated with quartiles of serum ALP levels among individuals with non-obesity (n=9,355).

**Variables**	PTB		SGA[§]		LGA[§]	
	OR (95 % CI)	***P*-value**	OR (95 % CI)	***P*-value**	OR (95%CI)	***P*-value**
Model 1[*]						
Q1 (<122 U/L)	1		1		1	
Q2 (122–148 U/L)	0.37 (0.30, 0.45)	<0.001	0.95 (0.78, 1.15)	0.589	1.21 (1.00, 1.46)	0.048
Q3 (149–182 U/L)	0.23 (0.18, 0.29)	<0.001	0.79 (0.65, 0.96)	0.018	1.21 (1.01, 1.46)	0.037
Q4 (>182 U/L)	0.19 (0.15, 0.25)	<0.001	0.80 (0.66, 0.97)	0.026	1.29 (1.08, 1.54)	0.006
P for trend		<0.001		0.011		0.013
Model 2[†]						
Q1 (<122 U/L)	1		1		1	
Q2 (122–148 U/L)	0.33 (0.27, 0.41)	<0.001	0.93 (0.77, 1.13)	0.480	1.42 (1.17, 1.72)	<0.001
Q3 (149–182 U/L)	0.19 (0.15, 0.25)	<0.001	0.77 (0.63, 0.94)	0.011	1.52 (1.26, 1.85)	<0.001
Q4 (>182 U/L)	0.15 (0.11, 0.19)	<0.001	0.76 (0.62, 0.94)	0.009	1.70 (1.40, 2.06)	<0.001
P for trend		<0.001		0.004		<0.001
Model 3[‡]						
Q1 (<122 U/L)	1		1		1	
Q2 (122–148 U/L)	0.32 (0.26, 0.41)	<0.001	0.89 (0.73, 1.09)	0.269	1.51 (1.23, 1.84)	<0.001
Q3 (149–182 U/L)	0.20 (0.15, 0.25)	<0.001	0.72 (0.58, 0.89)	0.002	1.63 (1.34, 2.00)	<0.001
Q4 (>182 U/L)	0.12 (0.09, 0.16)	<0.001	0.65 (0.52, 0.81)	<0.001	1.84 (1.50, 2.27)	<0.001
P for trend		<0.001		<0.001		<0.001

ALP – alkaline phosphatase, PTB – preterm birth, SGA – small for gestational age, LGA – large for gestational age, OR – odds ratio, CI – confidence interval, Q – quartile, BMI – body mass index, BP – blood pressure, hsCRP – high sensitive C-reactive protein

[*]Unadjusted.

[†]Adjusted for maternal age, BMI, parity, fetal gestational age, systolic and diastolic BP, pregnancy complications, assisted reproduction and fetal sex.

[‡]Adjusted for Model 2 variables plus blood lipids, hsCRP, liver and kidney function.

[§]Additionally corrected for fetal gestational age.

Table S5. ORs and 95 % CIs for adverse birth outcomes associated with quartiles of serum ALP levels among individuals with non-multipara (n=7,110)

**Variables**	PTB		SGA§		LGA§	
	OR (95 % CI)	***P*-value**	OR (95 % CI)	***P*-value**	OR (95%CI)	***P*-value**
Model 1*						
Q1 (<122 U/L)	1		1		1	
Q2 (122–148 U/L)	0.33 (0.26, 0.42)	<0.001	0.91 (0.73, 1.14)	0.425	1.30 (1.03, 1.63)	0.025
Q3 (149–182 U/L)	0.20 (0.15, 0.27)	<0.001	0.79 (0.63, 0.99)	0.037	1.35 (1.08, 1.68)	0.009
Q4 (>182 U/L)	0.20 (0.15, 0.26)	<0.001	0.79 (0.63, 0.98)	0.031	1.38 (1.11, 1.72)	0.004
P for trend		<0.001		0.021		0.011
Model 2†						
Q1 (<122 U/L)	1		1		1	
Q2 (122–148 U/L)	0.31 (0.24, 0.41)	<0.001	0.89 (0.71, 1.13)	0.347	1.46 (1.14, 1.85)	0.002
Q3 (149–182 U/L)	0.17 (0.13, 0.23)	<0.001	0.72 (0.57, 0.91)	0.005	1.65 (1.30, 2.10)	<0.001
Q4 (>182 U/L)	0.15 (0.11, 0.20)	<0.001	0.66 (0.52, 0.84)	0.001	1.91 (1.50, 2.43)	<0.001
P for trend		<0.001		<0.001		<0.001
Model 3‡						
Q1 (<122 U/L)	1		1		1	
Q2 (122–148 U/L)	0.30 (0.23, 0.40)	<0.001	0.87 (0.68, 1.10)	0.246	1.53 (1.19, 1.96)	0.001
Q3 (149–182 U/L)	0.18 (0.13, 0.25)	<0.001	0.68 (0.53, 0.86)	0.002	1.74 (1.35, 2.23)	<0.001
Q4 (>182 U/L)	0.13 (0.09, 0.18)	<0.001	0.58 (0.45, 0.75)	<0.001	1.97 (1.53, 2.53)	<0.001
P for trend		<0.001		<0.001		<0.001

ALP – alkaline phosphatase, PTB – preterm birth, SGA – small for gestational age, LGA – large for gestational age, OR – odds ratio, CI – confidence interval, Q – quartile, BMI– body mass index, BP – blood pressure, hsCRP – high sensitive C-reactive protein

*Unadjusted.

†Adjusted for maternal age, BMI, parity, fetal gestational age, systolic and diastolic BP, pregnancy complications, assisted reproduction and fetal sex.

‡Adjusted for Model 2 variables plus blood lipids, hsCRP, liver and kidney function.

§Additionally corrected for fetal gestational age.

Table S6. ORs and 95 % CIs for adverse birth outcomes associated with quartiles of serum ALP levels among individuals with non-pregnancy complications (n=9,676)

**Variables**	PTB		SGA[§]		LGA[§]	
	OR (95 % CI)	***P*-value**	OR (95 % CI)	***P*-value**	OR (95%CI)	***P*-value**
Model 1[*]						
Q1 (<122 U/L)	1		1		1	
Q2 (122–148 U/L)	0.33 (0.26, 0.41)	<0.001	0.90 (0.74, 1.09)	0.276	1.20 (1.02, 1.42)	0.031
Q3 (149–182 U/L)	0.22 (0.17, 0.28)	<0.001	0.78 (0.64, 0.96)	0.017	1.16 (0.98, 1.37)	0.083
Q4 (>182 U/L)	0.13 (0.10, 0.18)	<0.001	0.81 (0.66, 0.99)	0.043	1.20 (1.02, 1.42)	0.029
P for trend		<0.001		0.028		0.070
Model 2[†]						
Q1 (<122 U/L)	1		1		1	
Q2 (122–148 U/L)	0.32 (0.26, 0.40)	<0.001	0.83 (0.68, 1.03)	0.086	1.45 (1.21, 1.73)	<0.001
Q3 (149–182 U/L)	0.20 (0.15, 0.25)	<0.001	0.65 (0.53, 0.81)	<0.001	1.62 (1.35, 1.94)	<0.001
Q4 (>182 U/L)	0.11 (0.08, 0.15)	<0.001	0.65 (0.52, 0.81)	<0.001	1.91 (1.59, 2.30)	<0.001
P for trend		<0.001		<0.001		<0.001
Model 3[‡]						
Q1 (<122 U/L)	1		1		1	
Q2 (122–148 U/L)	0.31 (0.24, 0.39)	<0.001	0.79 (0.64, 0.98)	0.030	1.53 (1.27, 1.84)	<0.001
Q3 (149–182 U/L)	0.21 (0.16, 0.27)	<0.001	0.61 (0.49, 0.77)	<0.001	1.67 (1.38, 2.02)	<0.001
Q4 (>182 U/L)	0.11 (0.08, 0.16)	<0.001	0.57 (0.45, 0.72)	<0.001	1.99 (1.64, 2.42)	<0.001
P for trend		<0.001		<0.001		<0.001

ALP – alkaline phosphatase, PTB – preterm birth, SGA – small for gestational age, LGA – large for gestational age, OR – odds ratio, CI – confidence interval, Q – quartile, BMI – body mass index, BP – blood pressure, hsCRP – high sensitive C-reactive protein

[*]Unadjusted.

[†]Adjusted for maternal age, BMI, parity, fetal gestational age, systolic and diastolic BP, pregnancy complications, assisted reproduction and fetal sex.

[‡]Adjusted for Model 2 variables plus blood lipids, hsCRP, liver and kidney function.

[§]Additionally corrected for fetal gestational age.

Table S7. ORs and 95 % CIs for adverse birth outcomes associated with quartiles of serum ALP levels among individuals with full-term birth (n=11,004)

**Variables**	SGA		LGA	
	OR (95 % CI)	***P*-value**	OR (95 % CI)	***P*-value**
Model 1*				
Q1 (<122 U/L)	1		1	
Q2 (122–148 U/L)	0.97 (0.80, 1.17)	0.746	1.16 (0.99, 1.35)	0.063
Q3 (149–182 U/L)	0.85 (0.70, 1.03)	0.100	1.15 (0.99, 1.34)	0.071
Q4 (>182 U/L)	0.89 (0.74, 1.08)	0.249	1.20 (1.04, 1.40)	0.016
P for trend		0.170		0.034
Model 2†				
Q1 (<122 U/L)	1		1	
Q2 (122–148 U/L)	0.93 (0.76, 1.14)	0.486	1.27 (1.08, 1.50)	0.004
Q3 (149–182 U/L)	0.75 (0.61, 0.92)	0.005	1.43 (1.22, 1.68)	<0.001
Q4 (>182 U/L)	0.74 (0.60, 0.91)	0.004	1.67 (1.42, 1.97)	<0.001
P for trend		0.001		<0.001
Model 3‡				
Q1 (<122 U/L)	1		1	
Q2 (122–148 U/L)	0.91 (0.75, 1.12)	0.393	1.31 (1.11, 1.55)	0.002
Q3 (149–182 U/L)	0.73 (0.59, 0.90)	0.003	1.45 (1.23, 1.72)	<0.001
Q4 (>182 U/L)	0.69 (0.55, 0.85)	0.001	1.72 (1.45, 2.05)	<0.001
P for trend		<0.001		<0.001

ALP – alkaline phosphatase, SGA – small for gestational age, LGA – large for gestational age, OR – odds ratio, CI – confidence interval, Q – quartile, BMI – body mass index, BP – blood pressure, hsCRP – high sensitive C-reactive protein

*Unadjusted.

†Adjusted for maternal age, BMI, parity, fetal gestational age, systolic and diastolic BP, pregnancy complications, assisted reproduction and fetal sex.

‡Adjusted for Model 2 variables plus blood lipids, hsCRP, liver and kidney function.

Table S8. Subgroup analysis of **e**ffect modification of perinatal parameters (different categories) on association between ALP levels (Q4 vs. Q1–Q3) and PTB infants

**Variables**	Crude			Adjusted*		
	OR (95% CI)	*P* value	*P* for interaction	OR (95% CI)	*P* value	*P* for interaction
Age (years)						
< 35	0.38 (0.30, 0.47)	<0.001	0.769	0.30 (0.23, 0.39)	<0.001	0.859
≥ 35	0.50 (0.27, 0.92)	0.026		0.35 (0.17, 0.72)	0.005	
BMI (kg/m^2)						
< 30	0.39 (0.31, 0.49)	<0.001	0.741	0.30 (0.23, 0.40)	<0.001	0.943
≥ 30	0.39 (0.24, 0.65)	<0.001		0.19 (0.11, 0.32)	<0.001	
Parity						
No child	0.42 (0.33, 0.55)	0.154	0.227	0.31 (0.23, 0.41)	0.005	0.717
≥ 1 child	0.39 (0.27, 0.56)	0.833		0.33 (0.21, 0.49)	0.526	

ALP – alkaline phosphatase, Q – quartile, PTB – preterm birth, OR – odds ratio, CI – confidence interval, BMI – body mass index, BP – blood pressure, hsCRP – high sensitive C-reactive protein

*Adjusted for maternal age, BMI, parity, systolic and diastolic BP, pregnancy complications, assisted reproduction, fetal sex, and the levels of blood lipids, hsCRP, liver and kidney function, except for the covariate that was categorized.

Table S9. Subgroup analysis of **e**ffect modification of perinatal parameters (different categories) on association between ALP levels (Q4 vs. Q1–Q3) and SGA/LGA infants

**Variables**	Crude			Adjusted*		
	OR (95% CI)	***P*-value**	*P* for interaction	OR (95% CI)	***P*-value**	*P* for interaction
SGA						
Age (years)						
< 35	0.92 (0.79, 1.08)	0.297	0.551	0.78 (0.66, 0.93)	0.006	0.269
≥ 35	0.69 (0.40, 1.20)	0.193		0.56 (0.30, 1.05)	0.069	
BMI (kg/m^2)						
< 30	0.88 (0.76, 1.04)	0.130	0.605	0.81 (0.68, 0.96)	0.015	0.880
≥ 30	0.52 (0.33, 0.82)	0.005		0.36 (0.22, 0.59)	<0.001	
Parity						
No child	0.88 (0.74, 1.05)	0.154	0.853	0.76 (0.62, 0.92)	0.005	0.996
≥ 1 child	0.67 (0.51, 0.89)	0.005		0.66 (0.48, 0.89)	0.007	
PTB						
No	0.95 (0.82, 1.12)	0.556	0.917	0.80 (0.67, 0.95)	0.011	0.495
Yes	2.03 (1.17, 3.50)	0.012		0.93 (0.46, 1.87)	0.840	
LGA						
Age (years)						
< 35	1.07 (0.95, 1.21)	0.245	0.208	1.37 (1.20, 1.58)	<0.001	0.262
≥ 35	2.45 (1.85, 3.24)	<0.001		2.02 (1.47, 2.77)	<0.001	
BMI (kg/m^2)						
< 30	1.12 (0.98, 1.29)	0.092	0.428	1.27 (1.10, 1.48)	0.001	0.154
≥ 30	3.16 (2.57, 3.90)	<0.001		3.75 (2.99, 4.71)	<0.001	
Parity						
No child	1.13 (0.96, 1.32)	0.138	0.692	1.35 (1.14, 1.60)	<0.001	0.305
≥ 1 child	2.24 (1.88, 2.66)	<0.001		2.14 (1.76, 2.61)	<0.001	
PTB						
No	1.09 (0.97, 1.22)	0.155	0.444	1.35 (1.18, 1.53)	<0.001	0.259
Yes	1.06 (0.61, 1.86)	0.833		1.22 (0.65, 2.29)	0.526	

ALP – alkaline phosphatase, Q – quartile, SGA – small for gestational age, LGA – large for gestational age, OR – odds ratio, CI – confidence interval, BMI – body mass index, PTB – preterm birth, BP – blood pressure, hsCRP – high sensitive C-reactive protein

*Adjusted for maternal age, BMI, parity, systolic and diastolic BP, pregnancy complications, assisted reproduction, fetal gestational week, fetal sex, and the levels of blood lipids, hs-CRP, liver and kidney function, except for the covariate that was categorized.

Table S10. Accuracy of different serum indicators and models to predict PTB

Variables	AUC	95% CI	*P*-value‡	Best threshold	Specificity (%)	Sensitivity (%)	PPV (%)	NPV (%)
ALP (U/L)	0.697	0.677–0.717		126.5	73.14	57.83	14.24	95.74
Albumin (g/L)	0.640	0.683–0.719	< 0.001	35.55	64.59	57.13	11.07	95.13
Total protein (g/L)	0.631	0.612–0.651	< 0.001	61.95	64.16	55.01	10.59	94.87
Triglyceride (mmol/L)	0.604	0.583–0.624	< 0.001	2.89	73.56	42.98	10.75	94.57
Creatinine (μmol/L)	0.590	0.569–0.611	< 0.001	56.65	66.14	50.30	10.26	94.53
Total bilirubin (μmol/L)	0.583	0.563–0.604	< 0.001	6.05	73.95	40.33	10.70	94.12
hs-CRP (mg/L)	0.575	0.554–0.596	< 0.001	3.40	57.43	54.02	8.57	94.42
Urea nitrogen (mmol/L)	0.567	0.545–0.589	< 0.001	2.30	71.00	43.68	10.39	94.25
Total cholesterol (mmol/L)	0.564	0.543–0.585	< 0.001	6.36	49.15	60.75	8.13	94.41
ALT (U/L)	0.562	0.542–0.583	< 0.001	11.8	72.97	37.63	9.72	93.80
HDL–C (mmol/L)	0.544	0.523–0.565	< 0.001	1.48	76.55	30.68	8.84	93.71
LDL–C (mmol/L)	0.541	0.520–0.562	< 0.001	3.04	62.26	45.02	8.14	93.84
Direct bilirubin (μmol/L)	0.533	0.512–0.553	< 0.001	1.15	67.26	39.98	8.63	93.54
Model 1*	0.761	0.743–0.780		−2.32	82.31	57.90	18.80	96.51
Model 2†	0.809	0.791–0.826	< 0.001	−2.49	78.09	69.06	18.23	97.27

ALP – alkaline phosphatase, ALT – alanine aminotransferase, AUC – area under the curve, PTB – preterm birth, CI – confidence interval, HDL-C – high density lipoprotein cholesterol, hsCRP – high sensitive C-reactive protein, LDL-C – low density lipoprotein cholesterol, NPV – negative predictive value, PPV – positive predictive value.

*Model 1 included maternal age, height, weight, parity, blood pressure, pregnancy complications, assisted reproduction, foetal sex, blood lipids, liver and kidney function and hsCRP.

†Model 1 plus ALP.

‡*P*-values presented the significance of differences between ALP and 12 other variates or the difference between Model 1 and Model 2.
